# Type A Trichothecene Diacetoxyscirpenol-Induced Emesis Corresponds to Secretion of Peptide YY and Serotonin in Mink

**DOI:** 10.3390/toxins12060419

**Published:** 2020-06-25

**Authors:** Qinghua Wu, Kamil Kuca, Eugenie Nepovimova, Wenda Wu

**Affiliations:** 1 College of Life Science, Yangtze University, Jinzhou 434025, China; wqh212@hotmail.com; 2 Department of Chemistry, Faculty of Science, University of Hradec Kralove, 50003 Hradec Kralove, Czech Republic; eugenie.nepovimova@uhk.cz; 3 MOE Joint International Research Laboratory of Animal Health and Food Safety, College of Veterinary Medicine, Nanjing Agricultural University, Nanjing 210095, China

**Keywords:** mycotoxin, trichothecene, diacetoxyscirpenol, emesis, peptide YY, serotonin

## Abstract

The trichothecene mycotoxins contaminate cereal grains and have been related to alimentary toxicosis resulted in emetic response. This family of mycotoxins comprises type A to D groups of toxic sesquiterpene chemicals. Diacetoxyscirpenol (DAS), one of the most toxic type A trichothecenes, is considered to be a potential risk for human and animal health by the European Food Safety Authority. Other type A trichothecenes, T-2 toxin and HT-2 toxin, as well as type B trichothecene deoxynivalenol (DON), have been previously demonstrated to induce emetic response in the mink, and this response has been associated with the plasma elevation of neurotransmitters peptide YY (PYY) and serotonin (5-hydroxytryptamine, 5-HT). However, it is found that not all the type A and type B trichothecenes have the capacity to induce PYY and 5-HT. It is necessary to identify the roles of these two emetogenic mediators on DAS-induced emesis. The goal of this study was to determine the emetic effect of DAS and relate this effect to PYY and 5-HT, using a mink bioassay. Briefly, minks were fasted one day before experiment and given DAS by intraperitoneally and orally dosing on the experiment day. Then, emetic episodes were calculated and blood collection was employed for PYY and 5-HT test. DAS elicited robust emetic responses that corresponded to upraised PYY and 5-HT. Blocking the neuropeptide Y2 receptor (NPY2R) diminished emesis induction by PYY and DAS. The serotonin 3 receptor (5-HT3R) inhibitor granisetron totally restrained the induction of emesis by serotonin and DAS. In conclusion, our findings demonstrate that PYY and 5-HT have critical roles in DAS-induced emetic response.

## 1. Introduction

Trichothecenes are an important family of mycotoxins and regularly pollute food crops, animal feed and human foodstuffs. Predominantly produced by the mold fungus Fusarium, this family has over 150 mycotoxins and comprises type A to D groups of toxic sesquiterpene chemicals [1]. Due to the changes of climate and agricultural work in the world, the incidence rate of Fusarium head blight rises and leads to the elevation of trichothecene mycotoxins contamination [2]. This group of mycotoxins has long been linked with public health concern and elicits many adverse effects, such as emesis, nausea, anorexic effect, abdominal pain, growth suppression, diarrhea, hemorrhage, sicchasia and immunotoxicity [3]. In particular, the emetic potencies of trichothecenes are of particular concern from a public health perspective [4]. Interestingly, one of the trichothecenes was named “vomitoxin” (deoxynivalenol, DON), as a result of stimulating vomiting in swine [5]. 

Diacetoxyscirpenol (DAS) is considered to be one of the most toxic naturally occurring trichothecenes and belongs to the type A group of trichothecenes. Mainly produced by Fusarium langsethiae, poae and sambucinum, DAS contaminates various cereal grains (maize, barley, wheat, oats and sorghum), but also potato products, soybeans and coffee [6]. Usually, DAS co-occurs with many other mycotoxins in grains, in particular Fusarium mycotoxins, including type A and B trichothecenes, as well as zearalenone. According to the presence of DAS in grains and its high toxicity, the European Food Safety Authority (EFSA) indicated that this toxin had a potential risk for human and animal health [7]. However, the toxic effects and underlying mechanisms of DAS are not fully illustrated, due to the limitations in the available trial data. Similar to other trichothecenes, DAS exhibits a wide-ranging biological activity and toxicolgical effect, including an emetic effect. DAS elicits vomiting in several animal species, including ferret, dog, duck and swine, via oral, subcutaneous, intravenous and intraperitoneal injection (IP) [8,9,10,11,12,13]. Despite the importance of the emetic effect, the pathophysiological mechanisms of this adverse effect induction by DAS remain unclear.

Emetic response is a physiological process regulated by neurotransmitters, neuropeptides, vagal afferent neurons and the central pattern generator (CPG, also named “Vomiting Center”) in the hindbrain [14,15]. It refers to a protective response in prophylaxis against alimentary toxicosis; however, serious and persistent vomiting will disturb the balance of electrolyte and nutrient absorption resulted in severe influence on health [16]. Current investigations indicate that vomiting can occur through both peripheral and central pathways. In the peripheral pathway, the vomiting agents cause the secretion of the neurotransmitters by enterochromaffin (EC) cells in the intestine and bind their specific receptors located in the vagus afferent nerve. Then, it resulted in transmitting the stimuli to the nucleus tractus solitarius (NTS), activating the CPG and eventually evoking vomiting [17]. In the central pathway, the vomiting agents cause the secretion of the neurotransmitters by cerebric EC cells and bind their specific receptors located in area postrema, activating CPG and stimulating emetic response [18]. Neurotransmitters and neuropeptides play critical roles in pathophysiologic basis for vomiting, especially peptide YY (PYY) and serotonin (5-hydroxytryptamine, 5-HT). Some investigations suggest that PYY is an emetogenic peptide secreted by L cell (an important enteroendocrine cell type in the intestine), and have been reported to trigger vomiting in both human and animal species (such as dog and mink) [19,20]. Moreover, 5-HT has the capacity to mediate emetic episodes induction by chemotherapy drugs, such as cisplatin and carboplatin [21,22,23,24]. Cisplatin could elicit vomiting, as well as both 5-HT and PYY increases in canine [25].

To study emetic response caused by trichothecene mycotoxins, we have established a mink vomiting model [26]. Employing this vomiting bioassay, several type A and type B trichothecenes (DON, T-2 toxin, and HT-2 toxin) were proved to cause emetic responses, which have been linked to secretion of PYY and 5-HT [20,27]. Because we have found that not all the type A and type B trichothecenes have the capacity to induce PYY and 5-HT, it is necessary to identify the roles of these two emetogenic mediators on DAS-induced emesis. Here, we characterized the dose response and kinetics of emetic response to DAS, and clarify the roles of neurotransmitters PYY and 5-HT in emesis induction by DAS, using the mink model.

## 2. Results

### 2.1. Emetic Effects of IP and Oral Exposure to DAS

**Figure 1:**
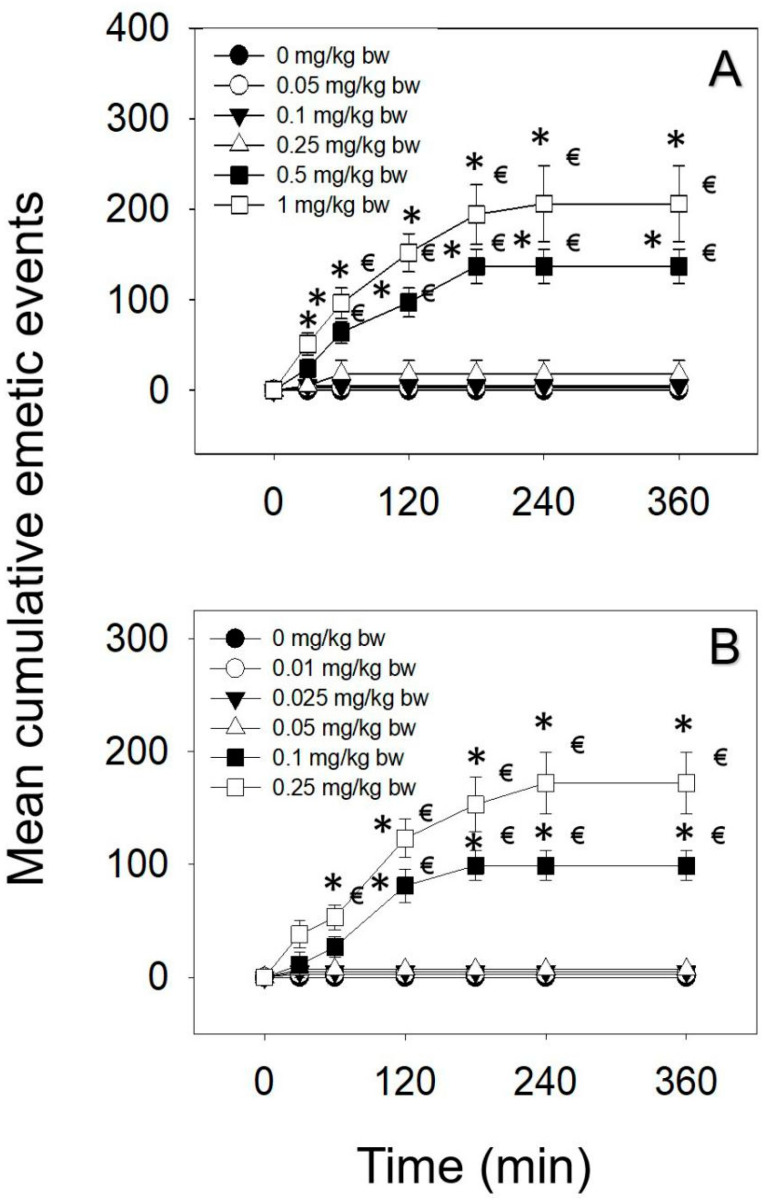
Mean cumulative emetic events in mink, following (**A**) IP and (**B**) oral exposure to DAS. Data represent mean ± SEM (n = 5/group). Two-way ANOVA using the Holm–Sidak method was used to assess significant differences in mean cumulative emetic events, as compared with the control. Symbols: * indicates a statistically significant difference in cumulative emetic episodes compared with the control (*p* < 0.05) and € indicates a statistically significant difference relative to the 0-min time point within a given dose (*p* < 0.05).

The emetic potencies of IP and oral dosing to DAS were exhibited in Table 1. Upon IP administration of DAS, 0.05 and 0.1 mg/kg bw had no effect (Figure 1A). The lowest dose at which DAS induced emesis following IP exposure was 0.25 mg/kg bw, with 20% of the mink responding. When mink intraperitoneally dosed with DAS at 0.5 and 1 mg/kg bw, emetic responses were detectable in 100% mink. The majority of emetic episodes occurred within 120 min, although new emetic episodes were recorded up to 180 and 240 min at the 0.5 and 1 mg/kg bw, respectively. The lowest dose at which DAS-induced emesis following oral exposure was 0.1 mg/kg bw, with 80% of the mink responding (Figure 1B). Moreover, 0.25 mg/kg bw DAS elicited 100% mink vomiting, and the majority of emetic episodes were observed within 120 min and continued up to 240 min.

### 2.2. DAS-Induced Emetic Effect Corresponds to Elevation of Plasma PYY and 5-HT

**Figure 2:**
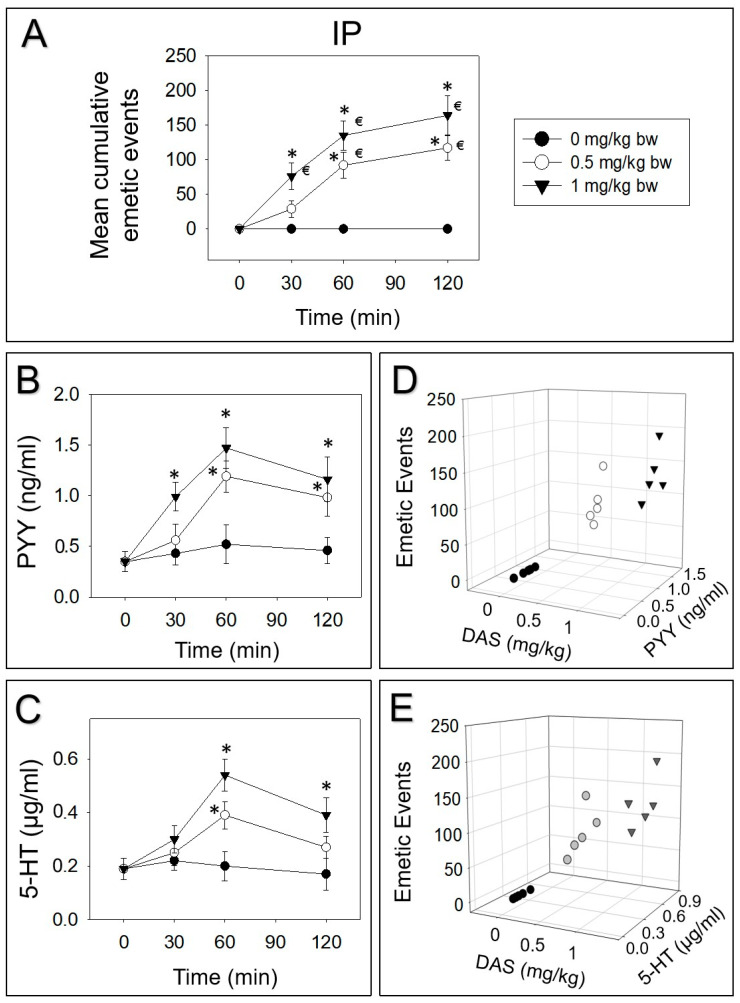
DAS-induced emetic effect corresponds to elevation of plasma peptide YY (PYY) and 5-HT, following IP exposure in mink. Data are mean ± SEM (n = 5/group). (**A**) Mean cumulative emetic events in mink following IP exposure to DAS. Kinetics of DAS-induced plasma (**B**) PYY and (**C**) 5-HT elevation. Relationship between emetic events and (**D**) PYY or (**E**) 5-HT levels at 60 min. Two-way ANOVA using the Holm–Sidak was used to analyze significant differences in mean cumulative emetic events and kinetics of plasma PYY and 5-HT in mink. Symbols: * indicates a statistically significant difference in mean cumulative emetic events and plasma PYY or 5-HT concentration, relative to the control at specific time point (*p* < 0.05) and € indicates a statistically significant difference in mean cumulative emetic events relative to the 0 min time point (*p* < 0.05). The Spearman rank-order correlation coefficient was used for correlation between emetic events and hormone levels (*p* < 0.05).

Mink were intraperitoneally and orally dosed with DAS. Then, PYY and 5-HT were measured over 120 min. IP dosing with 0.5 or 1 mg/kg bw DAS evoked 25%, 54% and 21% or 46%, 36% and 18% vomiting during 0–30, 30–60 and 60–120 min periods, respectively (Figure 2A). Plasma PYY was elevated at 30, 60 and 120 min or increased at 60 and 120 min, by receiving 0.5 or 1 mg/kg bw DAS, respectively (Figure 2B). Furthermore, 5-HT was increased at 60 and 120 min after exposure to 1 mg/kg bw DAS, while 0.5 mg/kg bw DAS only upraised 5-HT at 60 min (Figure 2C). Dose-dependent elevation of these two neurotransmitters corresponded to emetic episodes stimulated by DAS at 60 min (Figure 2D,E). 

**Figure 3:**
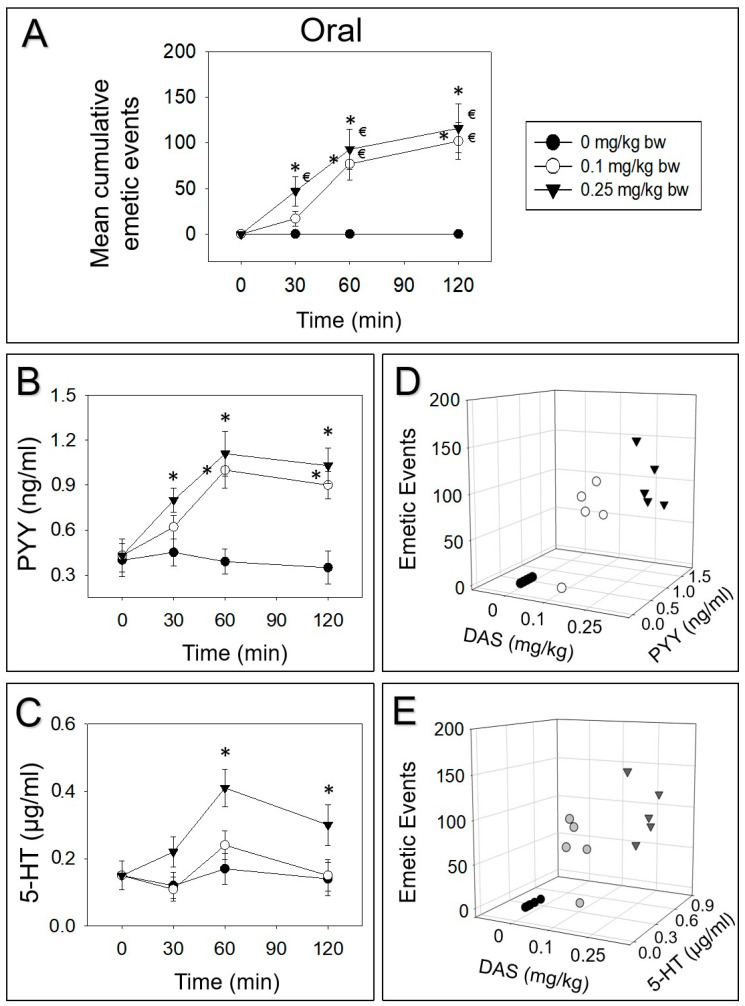
DAS-induced (**A**) emetic effect corresponds to the elevation of plasma PYY (**B**,**D**) and 5-HT (**C**,**E**) following oral exposure in mink. Experiment was carried out and data were assessed as depicted in Figure 2 legend.

Upon oral treatment of 0.1 or 0.25 mg/kg bw DAS, 17%, 58% and 25% or 41%, 39% and 20% vomiting occurred during 0–30, 30–60 and 60–120 min periods, respectively (Figure 3A). PYY was upraised at 30 min, peaked at 60 min and prominently appeared even at 120 min, following exposure to 0.25 mg/kg bw DAS (Figure 3B). Dosing with 0.1 mg/kg bw DAS markedly raised PYY at 60 and 120 min. Moreover, 5-HT was only increased after exposure to 0.25 mg/kg bw DAS at 60 and 120 min (Figure 3C). At 60 min, the elevation of PYY and 5-HT correlated with the emetic episodes evoked by DAS (Figure 3D,E).

### 2.3. Effects of NPY2 and 5-HT3 Receptor Antagonists on DAS-Induced Emesis

**Figure 4:**
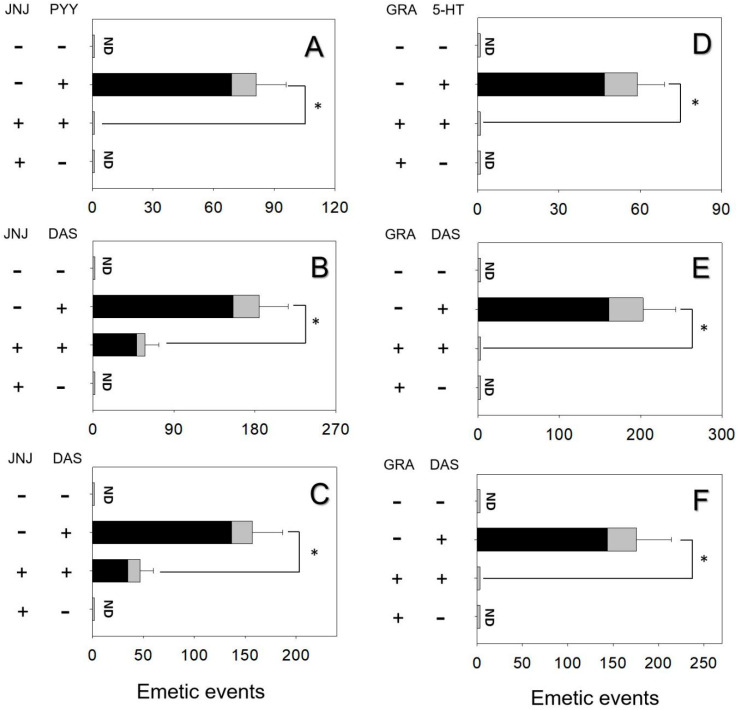
NPY2 receptor inhibitor JNJ-31020028 diminished emetic episodes induced by (**A**) PYY, (**B**) DAS following IP exposure, (**C**) DAS following oral exposure. Serotonin 3 receptor inhibitor granisetron abolished emetic episodes induced by (**D**) 5-HT, (**E**) DAS following IP exposure, (F) DAS following oral exposure. Emetic response comprises retching (black) and vomiting (gray) episodes. ND = not detected. Data represent mean ± SEM (n = 5/group). A one-way ANOVA using Holm–Sidak was used to assess significant differences between treatments and the respective controls. Symbols: * indicates statistically significant differences in emetic episodes (*p* < 0.05).

The effects of inhibiting the NPY2R in PYY- and DAS-induced vomiting were assessed. Mink dosing with PYY elicited 81 ± 15 emetic episodes (Figure 4A). Pretreated with the NPY2R inhibitor, JNJ-31020028 totally abolished emetic episodes induced by PYY. Giving JNJ-31020028 alone did not cause emetic episodes. Animals intraperitoneally and orally treated with DAS had 185 ± 32 and 157 ± 30 emetic episodes, respectively (Figure 4B and Figure 4C). Mink receiving JNJ-31020028 prior to DAS diminished 68% and 73% emetic episodes, respectively.

The effects of 5-HT3R inhibitor granisetron on vomiting caused by 5-HT and DAS were estimated. Mink dosing with 5-HT exhibited 59 ± 10 emetic episodes and the pretreatment of granisetron completely abolished emetic episodes (Figure 4D). Giving granisetron alone did not elicit emetic episodes, suggesting that there is no functional antagonism. The IP and oral administration of DAS exhibited 203 ± 40 and 176 ± 38 emetic episodes, respectively (Figure 4E and Figure 4F). Mink dosed with granisetron prior to DAS absolutely blocked emetic episodes.

## 3. Discussion

DAS is one of the most toxic type A trichothecenes and has been considered a potential risk for human and animal health by EFSA. The emetogenic potency refers to a hallmark of food toxicosis by DAS. However, the pathophysiological basis for this adverse effect is not fully understood. Previous investigations have elucidated that the trichothecenes could evoke robust emetic episodes in mink [26,28]. Although neurotransmitters 5-HT and PYY act critical roles in emesis induction by type A trichothecenes T-2 toxin and HT-2 toxin [20,27], it does not mean that DAS will have similar pathophysiological mechanisms unless verified. For instance, DON has the potential to induce PYY in the mouse. However, other type B trichothecenes, including 3-acetyldeoxynivalenol (3-ADON), 15-acetyldeoxynivalenol (15-ADON) and fusarenon X (FX), have no effect on eliciting PYY in the mouse [29]. This work is the first to relate the emetogenic potencies of DAS to these two neurotransmitters and elucidate the roles of NPY2R and 5-HT3R in emetic response induced by IP and orally dosing with DAS. The results revealed the novel findings that (1) emetic episodes induction following IP and orally dosing with DAS corresponded to the secretion of PYY and 5-HT in plasma; (2) PYY and 5-HT mediated emetic episodes via NPY2 and 5-HT3 receptors dependent mechanisms. 

Usually, mice and rat models are used for toxicology research, because they are smaller laboratory animals and convenient to be handled. However, both of them are rodents and lack an emetic reflex, suggesting that they are not suitable for emesis study [30]. Other smaller animal species with an emetic reflex used to study trichothecene-induced emesis include dogs and cats. Ethical issues of using companion species and maintenance costs for such studies present complications for their use as emesis models [31]. Mink (Neovison vison) is another small animal alternative to the above-mentioned models used in the emesis study. This species displays emetic behavior analogous to that reported for humans and thus are applicable to studying emetogenic compounds [32]. Notably, we have previously observed that several other trichothecenes stimulates emesis in mink, suggesting that this species is sensitive to this group of toxins [26]. The emetic potency data derived from mink emesis model are useful for predicting specific vomiting thresholds and risk assessments during DAS and other trichothecenes food poisoning.

Based on emesis incidence data, the no observed adverse effect level (NOAEL), lowest observed effect level (LOAEL) and median emetic dose (ED_50_) of DAS were determined (Table 2). The results reveal that DAS’s NOAEL, LOAEL and ED_50_ are less than its type A trichothecene congeners T-2 toxin and HT-2 toxin [27]. Interestingly, these values are not determined by toxicity. Instead, the absorption, distribution, metabolism, and excretion of toxin play vital roles. For instance, the observation of 100% emetic value for DAS in mink (0.5–1 mg/kg bw) is similar to the observation in ferret (1.5 mg/kg bw) by IP exposure [6]. Similar species in absorption, distribution, metabolism, and excretion of DAS following IP exposure might explain the similarity in this value. In addition to incidence, the emetogenic potential is specified by latency, duration and emetic episodes [33,34]. DAS elicited rapid (<60 min) and persistent (172–206 min) emetic episodes in mink, following IP and oral dosing. In ferret, 1.5 mg/kg bw DAS evoked emetic episodes at a latency of 22 min and duration of 120 min upon IP administration [6]. Bauer et al. [7] illustrated that upon oral exposure to DAS could be absorbed rapidly and reached a plasma peak level within 60 min, suggesting that the rapid absorption of DAS leads to fast latency to emesis. The emesis duration provides insight into the rate of elimination for DAS and the persistent duration indicates that DAS is eliminated slowly. DAS was reported to be metabolized and excreted within 24 h [7,8].

In this study, the secretion of PYY and 5-HT coincide with emetic episodes induced by DAS, suggesting that these two neurotransmitters play important roles in emetic response. Following IP and orally dosing with DAS, PYY was observed to significantly increase within 30–60 min and 5-HT was only upregulated by 60 min, indicating that the secretion of PYY occurred earlier than 5-HT. In support of our contention, intraperitoneally dosed to DON also exhibited a secretion of PYY (30–60 min) earlier than 5-HT (60 min) in mink [20]. One possible reason for the delayed elevation of plasma 5-HT might be its rapid metabolism. Following the secretion of 5-HT in the intestine, most of 5-HT will be transformed into 5-HIAA and impaired 5-HT levels in plasma [35,36]. Another possible reason is that PYY has the potential to affect the physiological function of 5-HT. When animals were pretreated by 5-HT3R inhibitor granisetron, it diminished not only PYY-induced emetic episodes, but also PYY levels [20,25]. Moreover, Kojima et al. [37] revealed that PYY is capable of evoking 5-HT secretion and it can be lessened by its receptor inhibitors, which is in support of our viewpoint. 

Here, DAS was demonstrated to evoke emetic episodes via NPY2 and 5-HT3 receptors dependent mechanisms. 5-HT3 and NPY2 receptors were demonstrated to contribute to emesis induction by DON in our prior investigation using the mink model [20]. In support of this contention, 5-HT was mentioned to involve in emesis induction through 5-HT3R by DON in swine [38]. Another research revealed that DAS had the capacity to stimulate emetic episodes and this adverse effect could be diminished by 5-HT3R inhibitor in ferrets [6]. Because the rodents are deficient in the vomiting center, the gastric emptying is considered to be a substitute for vomiting. Fioramonti and co-workers [39] implied that DON elicited delayed gastric emptying through 5-HT3R dependent mechanisms in rodents. Consistent with the function of NPY2R, PYY was mentioned to reduce gastric emptying in rodents via the NPY2R pathway [40]. DAS, as well as other trichothecenes, could induce 5-HT and PYY release in mice [41,42]. Moreover, 5-HT and PYY also have the potential to mediate anorexic responses evoked by trichothecenes, and the pretreatment of 5-HT3R and NPY2R inhibitors could diminish this adverse response [27,29,43], which also implicates the role of 5-HT3R and NPY2R in mediating 5-HT and PYY’s physiologic functions. 

## 4. Conclusions

**Figure 5:**
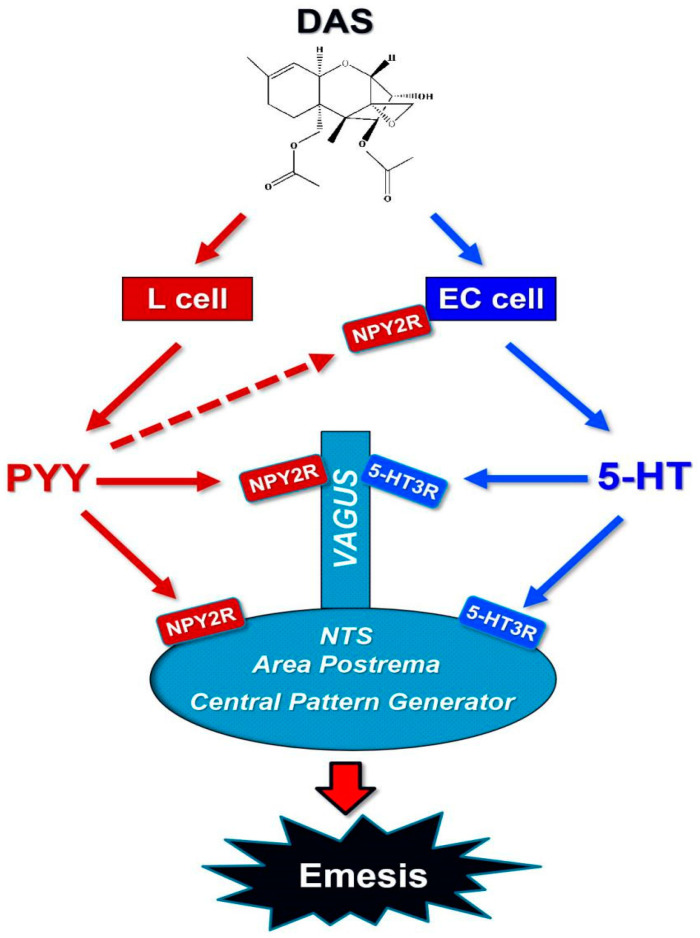
Putative mechanisms for vomiting induced by DAS.

In summary, the data presented herein show that PYY and 5-HT were robust increased by IP and orally dosing with DAS. Emetic effects induced by DAS were consistent with the elevation of these two neurotransmitters via the activation of NPY2R and 5-HT3R, as depicted in Figure 5. More investigations should be conducted on how other emetogenic modulators are involved in the emesis regulation of DAS through the peripheral and central pathways. Future research also needs to demonstrate the roles of other neurotransmitters and neuropeptides in the regulation of emesis. 

## 5. Materials and Methods 

### 5.1. Chemicals and Animals

DAS was obtained from Sigma-Aldrich (St. Louis, MI, USA) and tested by high performance liquid chromatography with a purity of more than 98%, as previously mentioned [3]. PYY, receptor inhibitors JNJ-31020028 and granisetron were obtained and prepared as previously mentioned [26]. Furthermore, 5-HT was gained from R&D systems and prepared in PBS. All guidelines for animal experiments followed the Institutional Animal Care and Use Committee at Nanjing Agricultural University (Certification No: SYXK (Su) 2011-0036; Date of approval: December 10th, 2017). One- or two-year-old female minks (Neovison vison) were purchased from Weifang Far East Breeding Co., Ltd. (Weifang, Shandong, China) and singly housed in cages at normal temperature (20–24 ℃), humidity (30–70%) and light/dark cycle (12 h). 

### 5.2. Experimental Design

The experimental design of emesis bioassay in mink was according to previous research [26]. Briefly, mink (n = 5/group) were fasted one day before the experiment and fed 30 min before treatment. At 9:00 h, mink were given 0, 0.05, 0.1, 0.25, 0.5, and 1 mg/kg bw DAS by IP injection, using a sterile 20-G, 2.54-cm needle, or orally gavaged with DAS at 0, 0.01, 0.025, 0.05, 0.1, and 0.25 mg/kg bw, using a sterile 16-G, 5-cm stainless steel gavage tube, respectively. After dosing, animals were returned to individual cages and monitored for emesis over the subsequent 6 h. Each individual retch or vomit was counted, as described previously [20].

The experimental design of peptide YY and serotonin study in mink was according to prior research [27]. Briefly, minks (n = 5/group) were fasted one day before experiment and feed 30 min before treatment. Then, animals were IP injected by 0, 0.5 and 1 mg/kg bw, or orally dosing with DAS at 0, 0.1 and 0.25 mg/kg bw in 100 µl PBS, respectively. Emetic episodes were calculated in 120 min. Following 0-, 30-, 60- and 120-min interval time, the anesthesia of the animals was employed by 10 mg/kg BW ketamin upon intramuscular dosing. Blood collection was employed through cardiac puncture using EDTA vacutubes and centrifuged 10 min (1000× *g*, 4 °C), to plasma for PYY (Phoenix Pharmaceuticals, Burlingame, CA, USA) and serotonin (Enzo Life Sciences, Plymouth Meeting, PA, USA) ELISA examination.

The general design for receptor inhibitor study was analogous to emesis bioassay, in addition to pre-dosing with inhibitor [20]. The dosage of inhibitors was chosen according to our preliminary studies. To estimate that the NPY2R inhibitor JNJ-31020028 could diminish DAS-induced emesis, animals were primarily treated with JNJ-31020028 at 10 mg/kg bw, following subcutaneous injection and feed 30 min. Then, 0.5 and 0.25 mg/kg bw DAS were dosed to mink by intraperitoneal and oral exposure, respectively. PYY at 0.01 mg/kg bw was intraperitoneally injected to mink as a positive control. Emetic responses were recorded over the subsequent 2 h. To evaluate that 5-HT3R granisetron has the potential to abate DAS-evoked emetic episodes, animals were primarily dosed with granisetron at 2 mg/kg bw, following intraperitoneal injection, and fed 30 min. After that, 0.5 and 0.25 mg/kg bw DAS were dosed to mink by intraperitoneal and oral administration, respectively. Mink was intraperitoneally injected by 5-HT at 0.05 mg/kg bw as positive control. Emetic responses were recorded over the subsequent 2 h.

### 5.3. Statistics

SigmaPlot 11 for Windows (Jandel Scientific; San Rafael, CA, USA) was used to analyze all the data, with the exception of emetic dose (ED), which was calculated with Proc Probit using SAS (Version 9.2, SAS, Cary, NC, USA). Significant differences were regarded at *p* < 0.05. Statistical methods were carried out as described in figure legends.

## Figures and Tables

**Table 1:** Comparison of emetogenic potentials upon intraperitoneal injection (IP) and oral exposure to diacetoxyscirpenol (DAS).

Exposure Route	Dose (mg/kg bw)	Incidence (Responding/Tested)	Latency (min) ^A,B^	Duration (min) ^A,B^	Retch (Times)	Vomit (Times)	Total (Times)
IP	0	0/5	-	-	0 ± 0	0 ± 0	0 ± 0
	0.05	0/5	-	-	0 ± 0	0 ± 0	0 ± 0
	0.1	0/5	-	-	0 ± 0	0 ± 0	0 ± 0
	0.25	1/5	42 ± 0 ^a^	3 ± 0 ^a^	7 ± 7	2 ± 2	9 ± 9
	0.5*	5/5	27 ± 4 ^a^	120 ± 13 ^b^	122 ± 11	15 ± 8	137 ± 19
	1*	5/5	18 ± 5 ^a^	189 ± 19 ^b^	181 ± 31	25 ± 11	206 ± 42
Oral	0	0/5	-	-	0 ± 0	0 ± 0	0 ± 0
	0.01	0/5	-	-	0 ± 0	0 ± 0	0 ± 0
	0.025	0/5	-	-	0 ± 0	0 ± 0	0 ± 0
	0.05	0/5	-	-	0 ± 0	0 ± 0	0 ± 0
	0.1*	4/5	29 ± 5 ^a^	97 ± 11 ^a^	87 ± 9	12 ± 4	99 ± 13
	0.25*	5/5	20 ± 2 ^a^	141 ± 23 ^a^	153 ± 16	19 ± 11	172 ± 27

^A^ Average of positive responders only. ^B^ If animals did not elicit emetic episodes, latency and duration are displayed as “-”. ^C^ Average of both non-responders and responders. Data are mean ± SEM. * indicate significant differences at *p* < 0.05 for incidence, retch, vomit and total emetic episodes. Different letters within a column indicate significant differences at *p* < 0.05.

**Table 2:** Summary of NOAELs, LOAELs, and ED50s for emetic effects of type A trichothecenes in mink.

Toxin (mg/kg BW)		IP			Oral	
	NOAEL^a^	LOAEL^b^	ED_5__0_^c^	NOAEL^a^	LOAEL^b^	ED_5__0_^c^
T-2^d^	0.01	0.05	0.05	0.005	0.05	0.02
HT-2^d^	0.01	0.05	0.05	0.005	0.05	0.02
DAS	0.1	0.25	0.3	0.05	0.1	0.07

^a^ NOAEL = no observed adverse effect level. ^b^ LOAEL = lowest observed adverse effect level. ^c^ ED50 = Dose causing emesis in 50% of the animals tested. ED50 values were determined using a Proc Probit model. ^d^ Data for T-2 and HT-2 were from Wu et al. [27]
